# Predictive Performances of Blood Parameter Ratios for Liver Inflammation and Advanced Liver Fibrosis in Chronic Hepatitis B Infection

**DOI:** 10.1155/2021/6644855

**Published:** 2021-04-10

**Authors:** Rongrong Ding, Xinlan Zhou, Dan Huang, Yanbing Wang, Xiufen Li, Li Yan, Wei Lu, Zongguo Yang, Zhanqing Zhang

**Affiliations:** ^1^Department of Hepatobiliary Medicine, Shanghai Public Health Clinical Center, Fudan University, Shanghai 201508, China; ^2^Department of Integrative Medicine, Shanghai Public Health Clinical Center, Fudan University, Shanghai 201508, China

## Abstract

**Objective:**

Blood parameter ratios, including neutrophil to lymphocyte ratio (NLR), platelet to lymphocyte ratio (PLR), and monocyte to lymphocyte ratio (MLR), have been reported that they are correlated to the progression of liver disease. This study is aimed at evaluating the predictive value of PLR, NLR, and MLR for liver inflammation and fibrosis in patients with chronic hepatitis B (CHB).

**Methods:**

We recruited 457 patients with CHB who underwent a liver biopsy and routine laboratory tests. Liver histology was assessed according to the Scheuer scoring system. The predictive accuracy for liver inflammation and fibrosis was assessed by receiver operating characteristics (ROC) analysis.

**Results:**

PLR and NLR presented significantly reverse correlation to liver inflammation and fibrosis. However, these correlations were not observed for MLR and liver histology. The AUROCs of PLR for assessing G2-3 and G3 were 0.676 and 0.705 with cutoffs 74.27 and 68.75, respectively. The AUROCs of NLR in predicting inflammatory scores G2-3 and G3 were 0.616 and 0.569 with cutoffs 1.36 and 1.85, respectively. The AUROCs of PLR for evaluating fibrosis stages S3-4 and S4 were 0.723 and 0.757 with cutoffs 79.67 and 74.27, respectively. The AUROCs of NLR for evaluating fibrosis stages S3-4 and S4 were 0.590 with cutoff 1.14.

**Conclusion:**

Although PLR has similar predictive power of progressive liver fibrosis compared with APRI, FIB-4, and GPR in CHB patients, it has the advantage of less cost and easy application with the potential to be widely used in clinical practice.

## 1. Introduction

Chronic hepatitis B (CHB) is still a serious public health problem worldwide that affects 257 million people all over the world [1]. It may cause progressive liver inflammation and fibrosis, cirrhosis, even end-stage liver disease, and hepatocellular carcinoma [2]. To reduce the burden of CHB, the early and accurate prediction of liver inflammation and fibrosis as well as timely antiviral treatment plays an important role for controlling the disease progression, which even decreases the morbidity and mortality of CHB-related end-stage liver disease [3].

At present, liver biopsy is considered the gold standard procedure to accurately diagnose the liver histological scores. However, some limitations of liver biopsy restrict its widely clinical application such as invasiveness, patient's discomfort, sampling error, potential risk of complications, and interobserver variability [4, 5]. In recent years, transient elasotgraphy (TE) has been introduced as a noninvasive, highly reproducible technique for assessment of liver fibrosis, especially in liver stages 3 and 4, which may reduce the need for liver biopsy [6–8]. Yet, some drawbacks such as expensive equipment and lack of trained operators limit the clinical application of TE especially in resource-limited environments. Therefore, many studies focus on developing simple and practical blood or serum noninvasive models, which are more accessible to the majority of the public [9].

WHO has recommended serum biomarkers including aspartate aminotransferase to the platelet ratio index (APRI) and four factor-based fibrosis index (FIB-4) as alternative methods for liver biopsy [10, 11]. However, the performances of APRI and FIB-4 for evaluation of liver fibrosis are still controversial [12, 13]. The gamma-glutamyl transpeptidase-to-platelet ratio (GPR) is more accurate than APRI and FIB-4 to estimate liver fibrosis in West Africa cohorts with CHB, but it was not superior to APRI and FIB-4 in a French cohort [14]. Other studies also have not observed the advantages of GPR [15, 16].

Platelet-to-lymphocyte ratio (PLR), neutrophil-to-lymphocyte ratio (NLR), and monocyte-to-lymphocyte ratio (MLR) are low-cost and easy to reproducible calculation even in simple laboratory conditions. Several researches have indicated that these ratios have values to be used as predictors for prognosis or inflammation in patients with cardiovascular disease, autoimmune diseases, and mood disorders [17–19]. Recently, Zhou et al. [20] reported that PLR and NLR were related to the disease severity in CHB patients. A study by Lu et al. [21] indicated that PLR could be useful in predicting liver advanced fibrosis and cirrhosis. Another recent study showed MLR and NLR may be potential prognostic markers for predicting poor outcome in patients with CHB-related liver failure. Nevertheless, the changes of these lymphocytes ratio models at different liver histological stages have been rarely studied. Therefore, we evaluated the clinical significances of the above six blood markers in predicting liver inflammation and fibrosis in CHB patients.

## 2. Materials and Methods

### 2.1. Ethic Statement

The study protocol and informed consent documents were reviewed and approved by the Ethics Committee of Shanghai Public Health Clinical Center, Fudan University. All these chronic hepatitis patients provided written informed consent before participating in this study.

### 2.2. Study Population

A total of 457 consecutive treatment-naïve patients with CHB who underwent percutaneous liver biopsy at Shanghai Public Health Clinical Center, Fudan University, from December 2015 to January 2018 were retrospectively studied. The inclusion criteria were clinical history of CHB or HBsAg positive for more than 6 months, age ≥ 18 years, and discontinuation of potential lowering serum transaminase agents for at least 2 weeks prior to routine laboratory tests. The exclusion criteria were history of antiviral therapy, HCV and HIV coinfection, overt alcoholic or nonalcoholic fatty liver disease, autoimmune liver disease, hereditary metabolic liver diseases, decompensated cirrhosis, and pregnancy.

### 2.3. Liver Biopsy

Percutaneous liver biopsy was performed using a 16 G needle under ultrasound guidance. Liver samples with a minimum length of 1.5 cm and at least 6 complete portal tracts were considered suitable for liver histological scoring [22, 23]. Liver histology was analyzed by two experienced pathologists who were blinded to other clinical and laboratory data and classified according to the Scheuer scoring system [24].

### 2.4. Routine Laboratory Parameters

Fasting blood samples were obtained within a week of liver biopsy. Platelets and other blood cells were counted using a Sysmex-XT 4000i automated hematology analyzer. Serum alanine transaminase (ALT), aspartate aminotransferase (AST), alkaline phosphatase (ALP), gamma-glutamyl transfetase (GGT), bilirubin, albumin, and other serum biochemical parameters were measured using an Architectc16000 automatic biochemical analysis system. HBV DNA was quantified by real-time PCR (ABI 7500; Applied Biosystems, Foster City, CA, USA). Serum HBeAg was measured using a chemiluminescence microparticle immunoassay Abbot Architect 12000 automated analyzer and auxiliary reagents.

### 2.5. Formulas

The formulas for PLR, NLR, MLR, APRI, FIB-4, and GPR are as follows: PLR = platelet count (10^9^/L)/lymphocyte count (10^9^/L), NLR = neutrophil count (10^9^/L)/lymphocyte count (10^9^/L), MLR = monocyte count (10^9^/L)/lymphocyte count (10^9^/L), APRI = (AST (U/L)/ULN of AST)/platelet count (10^9^/L) × 100, FIB − 4 = (age (years) × AST (U/L))/(platelet count (10^9^/L) × (ALT (U/L))^1/2^), and GPR = (GGT (U/L)/ULN of GGT)/platelet count (10^9^/L) × 100.

### 2.6. Statistical Analysis

Statistical analysis was performed using IBM SPSS Statistics version 26.0 (SPSS Inc., Chicago, USA) and *R* 4.0.4 (http://www.R-project.org). Continuous variables were given as the median (interquartile range, IQR) and compared using the independent Mann–Whitney test. Categorical variables were given as proportions and compared by the Chi-square test. Correlations were evaluated by Spear's correlation coefficient for continuous variables. The performances of serum models for predicting liver histological scores were assessed by receiver operating characteristic (ROC) curve analyses and the area under the ROC curves (AUROCs). The DeLong *Z* test was used to compare the AUROC of the serum models. A two-sided *P* < 0.05 was considered statistically significant difference.

## 3. Results

### 3.1. Patient Clinical Profiles

The baseline clinical characteristics of enrolled patients are described in Table 1. The average age of the enrolled patients was 37 years. Most of them were men (66.5%) and HBeAg positive (61.1%). The distribution of liver inflammatory activities was 201 patients with G0-1 and 256 with G2-3, respectively. The distribution of fibrosis stages was 237 patients with S3-4 and 220 with S4, respectively. Compared with patients in G0-1, patients in G2-3 had higher ALT, AST, GGT, globulin, and HBV DNA, lower albumin, WBC counts, and platelet counts. Similarly, patients with S4 had higher ALT, AST, GGT, TBil, globin, APRI, FIB-4, and GPR, and lower albumin, platelet counts, PLR, and NLR. No significant differences were seen in MLR between patients with G0-1 and G2-3 or patients with S3-4 and S4.

### 3.2. Serological Models and Liver Histological Scores

**Figure 1:**
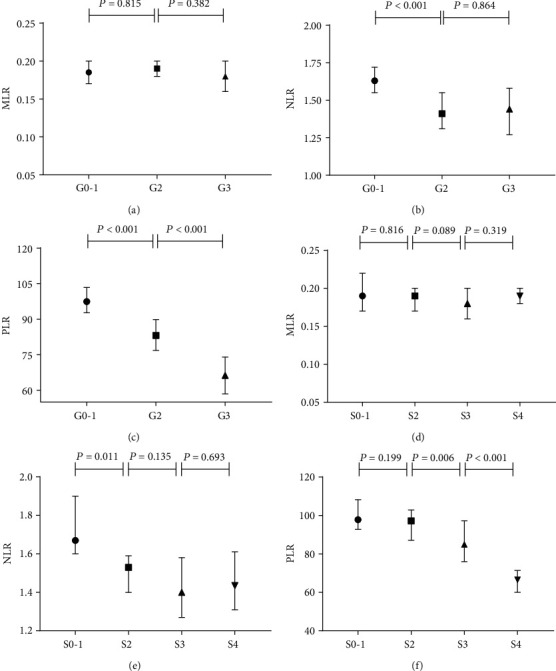
Medians in subgroups classified by inflammation grades and fibrosis stages (Scheuer scoring system). The medians of MLR (a) in G0-1, G2, and G3 were 0.18, 0.19, and 0.18, respectively; the median of NLR (b) in G0-1, G2, and G3 were 1.63, 1.41, and 1.44, respectively, and the median of PLR (d) in G0-1, G2, and G3 were 97.46, 83.12, and 66.23, respectively. As for liver fibrosis, the medians of MLR (d) in S0-1, S2, S3, and S4 were 0.19, 0.19, 0.18, and 0.19, respectively; the medians of NLR (e) in S0-1, S2, S3, and S4 were 1.67, 1.53, 1.40, and 1.44, respectively, and the medians of PLR (f) in S0-1, S2, S3, and S4 were 97.85, 97.21, 84.97, and 66.50, respectively.

The associations of PLR, NLR, and MLR with liver histopathology were further analyzed (Figures 1(a)–1(f)). As the liver histological scores increased, the PRL decreased. Spearman's correlation analysis presented that PLR (*r* = −0.372), NLR (*r* = −0.194), APRI (*r* = 0.586), FIB-4 (*r* = 0.470), and GPR (*r* = 0.601) were significantly correlated with liver inflammatory activities. As for liver fibrosis, PLR (*r* = −0.414), NLR (-0.172), APRI (*r* = 0.446), FIB-4 (0.412), and GPR (*r* = 0.506) were significantly correlated with fibrosis stages (Table 2).

### 3.3. Performances of PLR, NLR, APRI, FIB-4, and GPR for the Evaluation of Liver Inflammation

**Figure 2:**
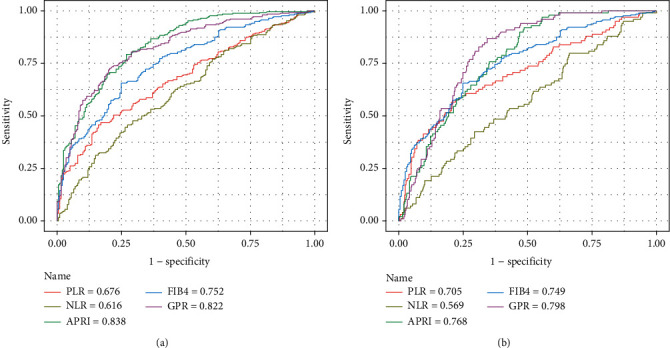
ROC comparison of PLR, NLR, APRI, FIB-4, and GPR for predicting liver inflammation. (a) ROC comparison for predicting G2-3. (b) ROC comparison for predicting G3.

The ROC curves of PLR, NLR, APRI, FIB-4, and GPR for predicting liver inflammation in patients with CHB are shown in Figure 2. The diagnostic performances of the different markers are demonstrated in Table 3.

The AUROCs of PLR for assessing inflammatory scores G2-3 and G3 were 0.676 (95%CI = 0.631 − 0.719) and 0.705 (95%CI = 0.661 − 0.747) with cutoffs 74.27 and 68.75, respectively. The AUROCs of NLR in predicting inflammatory scores G2-3 and G3 were 0.616 (95%CI = 0.570 − 0.661) and 0.569 (95%CI = 0.523 − 0.615) with cutoffs 1.36 and 1.85, respectively.

For the prediction of the inflammatory score G2-3, AUROC of PLR was better than that of NLR, but was significantly lower than APRI (0.838, 95%CI = 0.801 − 0.870, *P* < 0.0001), FIB-4 (0.752, 95%CI = 0.710 − 0.791, *P* = 0.009), and GPR (0.822, 95%CI = 0.784 − 0.856, *P* < 0.0001). Regarding the prediction of the inflammatory score G3, although AUROC of PLR was still lower than GPR (0.798, 95%CI = 0.759 − 0.834, *P* = 0.009), it was superior to that of NLR and comparable with APRI (0.768, 95%CI = 0.727 − 0.806, *P* = 0.094) and FIB-4 (0.749, 95%CI = 0.706 − 0.788, *P* = 0.269).

### 3.4. Performances of PLR, NLR, APRI, FIB-4, and GPR for the Evaluation of Liver Fibrosis

**Figure 3:**
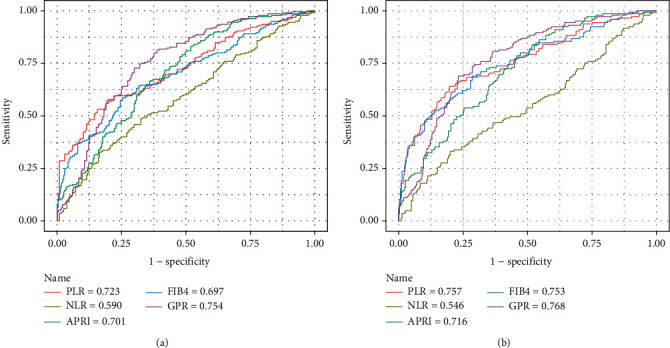
ROC comparison of PLR, NLR, APRI, FIB-4, and GPR for predicting liver fibrosis. (a) ROC comparison for predicting S3-4. (b) ROC comparison for predicting S4.

The ROC curves of PLR, NLR, APRI, FIB-4, and GPR for predicting liver fibrosis in patients with CHB are shown in Figure 3. The diagnostic performances of the different markers are demonstrated in Table 4.

The AUROCs of PLR for evaluating fibrosis stages S3-4 and S4 were 0.723 (95%CI = 0.697 − 0.764) and 0.757 (95%CI = 0.715 − 0.796) with cutoffs 79.67 and 74.27, respectively. The AUROCs of NLR for evaluating fibrosis stages S3-4 and S4 were 0.590 (95%CI = 0.544 − 0.636) with cutoff 1.14. There was no statistically significant difference of the AUROC of NLR for staging S4.

For staging fibrosis S3-4, AUROC of PLR was higher than that of NLR and was comparable with APRI (0.701, 95%CI = 0.657 − 0.743, *P* = 0.448), FIB-4 (0.697, 95%CI = 0.654 − 0.739, *P* = 0.359), and GPR (0.754, 95%CI = 0.712 − 0.793, *P* = 0.272). Similarly, to stage S4, AUROC of PLR was comparable with APRI (0.716, 95%CI = 0.672 − 0.757, *P* = 0.168), FIB-4 (0.753, 95%CI = 0.711 − 0.792, *P* = 0.883), and GPR (0.768, 95%CI = 0.726 − 0.806, *P* = 0.718).

## 4. Discussion

Early diagnosis and accurate evaluation of liver inflammation and fibrosis are not only important for control of the progression of the disease but also for the treatment of chronic HBV infection [3]. Because of the limitations of liver biopsy, many researchers have tried to propose noninvasive ideal procedures to evaluate liver inflammation and fibrosis which should be simple, low-cost, repeatable, and accurate [13]. In the present study, we evaluated and compared the performances of PLR and NLR with APRI, FIB-4, and GPR, using histology as reference.

This study observed the presence of statistically significantly reverse correlations between the PLR values and liver pathological scores. PLR had good performance to stage advanced fibrosis (S3-4) with an AUROC of 0.72 at a cutoff of 9.67 and cirrhosis (S4) with an AUROC of 0.76 at a cutoff of 74.27. These results were consistent with previous study [21]. Lu et al. [21] reported the AUROC of PLR for advanced fibrosis was 0.7 at a cutoff of 73.27 and considered it as an easily available and cheap marker for evaluation of liver fibrosis and cirrhosis. Additionally, PLR performed comparably to classical serum biomarkers including APRI, FIB-4, and GPR. However, as for liver inflammation, the AUROCs of PLR for detecting significant inflammation (G2-3) and sever inflammation (G3) were 0.68 and 0.71 with cutoff at 74.27 and 68.75, respectively. In comparison with APRI and GPR, performance of PLR showed significantly lower AUROC for both assessment of G2-3 and G3. The PLR is a comprehensive indicator of changes in immune status during disease because it is calculated as the platelet count/lymphocyte count and accounts for variations in platelet and lymphocyte numbers [13]. The PLR value is also related to the progression and prognosis of sudden deafness, vestibular neuritis, cardiovascular disease, and thrombosis-related diseases [25–27]. Moreover, PLR values could serve as a predictor for the prognosis and progression of viral hepatitis and hepatocellular carcinoma [20, 28–30].

NLR also could be an important marker of systemic inflammation with the advantages including cost effect, ready availability, and easy calculation. It integrates two immune pathways that neutrophils indicate persistent inflammation and lymphocytes indicate the regulatory pathway [31]. NLR has been associated to various inflammations and cardiovascular diseases [17, 32, 33]. In addition, a higher NLR could predict poor prognosis in many cancers, hepatocellular, pancreatic, gastric cancers, and non-small-cell lung [34–37]. Recently, a few studies have estimated the predictive power of NLR in patients with liver fibrosis and liver cirrhosis. In our study, NLR also manifested a statistically significant reverse correlation with liver fibrosis. These observations were consistent with previous studies [38, 39]. They showed that there was a possibility to use NLR as a predictive factor of liver fibrosis in CHB patients. However, in comparison with PLR, APRI, GPR, and FIB-4, performances of NLR to predict liver inflammation and fibrosis showed significantly lower AUROCs. Similar to our findings, a study by Huang et al. [40] demonstrated that the AUROCs of NLR for diagnosing advanced liver fibrosis and cirrhosis were 0.41 (95%CI = 0.34 − 0.48) and 0.44 (95%CI = 0.37 − 0.52). These results indicated that NLR did not sufficiently reflect the inflammation and the amount of the accumulated fibrous tissue in the liver.

Moreover, we validated the performance of APRI, GPR, and FIB-4 in diagnosing liver inflammation and fibrosis. The results showed that these classical noninvasive indexes were potential useful for diagnosis of liver inflammation and fibrosis. For liver inflammation, APRI and GPR were superior to FIB-4 for predicting G2-3, while the performances of the three markers were comparable for predicting G3. However, regarding liver fibrosis, GPR was superior to APRI and FIB-4. Similar to our study, a recent study by Wu et al. [12] showed that the AUROCs of APRI, FIB-4, and GPR for predicting ≥ G2 were 0.73, 0.70, 0.73, and for G3 were 0.86, 0.71, and 0.88, respectively. Another study showed lower AUROCs of the three markers for predicting liver inflammation that could be explained by the selection bias excluding CHB patients with the ALT level higher than two times of the ULN [41]. Additionally, our research and previous study confirmed that compared with APRI and FIB-4, GPR was more effective in evaluation of liver fibrosis [11, 14, 42].

One limitation of this study was a single-centre retrospective study; thus, the results should be further confirmed in multicentre prospectively researches with large-scale populations. Furthermore, we could not evaluate the potential correlation between these markers with liver inflammation and fibrosis in patients with concomitant CHB and nonalcoholic fatty liver disease. It is reported that the prevalence of nonalcoholic fatty liver disease was 20% in patients with CHB [43].

In conclusion, the present study demonstrates that PLR is a potentially useful noninvasive marker for predicting advanced fibrosis and cirrhosis. Although PLR has similar predictive power of progressive liver fibrosis compare with APRI, FIB-4, and GPR in CHB patients, it has the advantage of less cost and easy application with the potential to be widely used in clinical practice. However, PRL does not show advantages in prediction of liver inflammation compared to APRI, FIB-4, and GPR.

## Figures and Tables

**Table 1:** Clinical characteristics of the study patients.

Variables	Total (*n* = 457)	Inflammatory activity			Fibrosis stage		
		G0-1 (*n* = 201)	G2-3 (*n* = 256)	*P* value	S0-2 (*n* = 237)	S3-4 (*n* = 220)	*P* value
Age, years	37 (30-44)	37 (31-46)	36 (30-44)	0.103	36 (30-44)	37 (31-45)	0.410
Male, *n* (%)	284 (66.5)	129 (64.2)	175 (68.4)	0.347	152 (64.1)	152 (69.1)	0.262
Serological parameters							
HBeAg positive, *n* (%)	279 (61.1)	93 (46.3)	186 (72.7)	<0.001	132 (55.7)	147 (66.8)	0.015
HBV DNA, log_10_ IU/ml)	6.3 (4.1-7.3)	4.9 (3.0-7.3)	6.6 (5.3-7.4)	<0.001	6.4 (3.6-7.5)	6.1 (4.5-7.0)	0.543
ALT, U/L	63.0 (32.0-163.0)	37.0 (18.0-71.0)	106.5 (48.3-286.0)	<0.001	53.0 (22.0-147.5)	74.0 (38.3-195.2)	<0.001
AST, U/L	46.0 (26.0-98.0)	28.0 (20.0-45.5)	75.5 (42.0-170.0)	<0.001	36.0 (22.0-89.5)	58.0 (33.3-114.8)	<0.001
GGT, U/L	38.0 (19.0-79.5)	22.0 (14.5-39.0)	63.0 (32.3-112.7)	<0.001	26.0 (15.0-52.0)	55.5 (31.0-98.8)	<0.001
TBil, *μ*mol/L	15.1 (11.1-20.7)	14.3 (11.1-17.9)	15.9 (11.3-22.0)	0.007	14.2 (10.6-18.2)	16.7 (11.4-23.8)	<0.001
Albumin, g/L	41.5 (38.5-44.0)	42.8 (40.5-45.6)	40.0 (37.0-43.5)	<0.001	42.2 (39.7-45.0)	40.3 (37.3-43.6)	<0.001
Globulin, g/L	29.0 (26.9-33.0)	29.0 (26.0-32.0)	30.0 (27.0-33.7)	0.001	29.0 (26.0-32.0)	30.0 (27.0-33.0)	0.012
WBC, ×10^9^/L	5.1 (4.1-6.1)	5.4 (4.1-6.3)	5.0 (4.2-6.01)	0.029	5.3 (4.3-6.2)	4.9 (4.1-6.1)	0.066
Neutrophil count, ×10^9^/L	2.7 (2.1-3.5)	3.0 (2.2-3.8)	2.5 (2.0-3.2)	<0.001	2.8 (2.3-3.6)	2.6 (1.9-3.3)	0.001
Monocyte count, ×10^9^/L	0.3 (0.3-0.4)	0.3 (0.3-0.4)	0.3 (0.3-0.4)	0.239	0.3 (0.3-0.4)	0.3 (0.3-0.4)	0.439
Lymphocyte count, ×10^9^/L	1.8 (1.4-2.1)	1.7 (1.4-2.1)	1.8 (1.4-2.2)	0.229	1.8 (1.5-2.1)	1.8 (1.4-2.2)	0.409
Platelet, ×10^9^/L	157 (124-192)	177 (148-201)	142 (110-177)	<0.001	177 (150-202)	139 (99-166)	0.001
Serological indexes							
PLR	87.54 (66.49-111.22)	97.26 (79.74-120.49)	77.05 (58.49-101.86)	<0.001	97.46 (80.41-122.99)	71.46 (54.30-98.11)	<0.001
NLR	1.55 (1.17-2.02)	1.63 (1.31-2.21)	1.43 (1.08-1.82)	<0.001	1.59 (1.28-2.10)	1.42 (1.05-1.86)	0.001
MLR	0.19 (0.15-2.02)	0.18 (0.14-0.25)	0.19 (0.15-0.25)	0.985	0.19 (0.15-0.25)	0.19 (0.14-0.25)	0.147
APRI	0.81 (0.40-1.65)	0.41 (0.27-0.75)	1.34 (0.73-2.37)	<0.001	0.53 (0.29-1.23)	1.09 (0.61-2.12)	<0.001
FIB-4	1.41 (0.97-2.39)	1.09 (0.80-1.52)	1.81 (1.24-3.38)	<0.001	1.21 (0.87-1.67)	1.85 (1.19-3.51)	<0.001
GPR	0.25 (0.12-0.66)	0.13 (0.08-0.23)	0.49 (0.23-1.10)	<0.001	0.15 (0.08-0.31)	0.43 (0.23-0.95)	0.001

ALT: alanine aminotransferase; AST: aspartate aminotransferase; GGT: gamma-glutamyl transpeptidase; TBil: total bilirubin; WBC: white blood cell.

**Table 2:** Correlation between the noninvasive indexes and liver pathology score.

Indexes	Inflammatory activity		Fibrosis stage	
	*r*	*P* value	*r*	*P* value
PLR	-0.372	<0.001	-0.414	<0.001
NLR	-0.194	<0.001	-0.172	<0.001
MLR	-0.022	0.648	-0.062	0.189
APRI	0.586	<0.001	0.446	<0.001
FIB-4	0.470	<0.001	0.412	<0.001
GPR	0.601	<0.001	0.506	<0.001

**Table 3:** Predictive performance of serological indexes for assessing liver inflammatory.

	AUROC (95% CI)	*P* value	Cut-off	Se (%)	Sp (%)	PPV (%)	NPV (%)	Accuracy (%)	^∗^ *P* value
PLR									
G2-3	0.676 (0.631-0.719)	<0.0001	74.27	46.9	83.0	54.2	78.5	62.6	—
G3	0.705 (0.661-0.747)	<0.0001	68.75	56.6	79.6	54.2	83.9	72.9	—
NLR									
G2-3	0.616 (0.570-0.661)	<0.0001	1.36	45.7	72.6	41.7	75.7	57.3	0.044
G3	0.569 (0.523-0.615)	0.0337	1.85	79.8	33.8	34.0	79.5	57.8	0.0001
APRI									
G2-3	0.838 (0.801-0.870)	<0.0001	0.65	79.3	73.1	55.8	89.2	76.4	<0.0001
G3	0.768 (0.727-0.806)	<0.0001	0.68	91.9	51.4	44.8	93.7	60.6	0.094
FIB-4									
G2-3	0.752 (0.710-0.791)	<0.0001	1.48	65.6	75.1	53.1	83.6	69.8	0.009
G3	0.749 (0.706-0.788)	<0.0001	1.53	76.8	62.3	46.6	86.2	58.6	0.269
GPR									
G2-3	0.822 (0.784-0.856)	<0.0001	0.25	72.7	79.6	60.4	87.2	75.5	<0.0001
G3	0.798 (0.759-0.834)	<0.0001	0.29	86.9	65.6	52.0	92.1	70.7	0.009

AUROC: area under ROC; Se: sensitivity; Sp: specificity; PPV: positive predictive value; NPV: negative predictive value. ^∗^Compared with PLR.

**Table 4:** Predictive performance of serological indexes for assessing liver fibrosis.

	AUROC (95% CI)	*P* value	Cut-off	Se (%)	Sp (%)	PPV (%)	NPV (%)	Accuracy	^∗^ *P* value
PLR									
S3-4	0.723 (0.697-0.764)	<0.0001	79.67	59.6	78.0	53.7	81.8	68.9	—
S4	0.757 (0.715-0.796)	<0.0001	74.27	64.2	80.4	58.4	83.9	75.1	—
NLR									
S3-4	0.590 (0.544-0.636)	0.0007	1.14	32.7	83.5	46.0	74.3	59.1	<0.0001
S4	0.546 (0.499-0.592)	0.123	—	—	—	—	—	—	—
APRI									
S3-4	0.701 (0.657-0.743)	<0.0001	0.51	83.2	48.1	40.7	87.0	64.7	0.448
S4	0.716 (0.672-0.757)	<0.0001	0.66	80.0	52.8	42.1	86.1	61.3	0.168
FIB-4									
S3-4	0.697 (0.653-0.739)	<0.0001	1.61	58.6	74.7	49.8	80.8	66.5	0.359
S4	0.753 (0.711-0.792)	<0.0001	1.65	70.0	71.5	50.9	84.3	69.6	0.883
GPR									
S3-4	0.754 (0.712-0.793)	<0.0001	0.25	72.7	70.0	51.0	85.7	70.2	0.272
S4	0.768 (0.726-0.806)	<0.0001	0.38	69.0	76.9	56.2	85.3	74.0	0.718

AUROC: area under ROC; Se: sensitivity; Sp: specificity; PPV: positive predictive value; NPV: negative predictive value. ^∗^Compared with PLR.

## Data Availability

Datasets of the current study are available from the corresponding authors on reasonable request.
